# BRD4 regulation of PIM1 identifies a novel therapeutic vulnerability in acute megakaryoblastic leukemia

**DOI:** 10.1186/s12935-026-04377-1

**Published:** 2026-06-17

**Authors:** Ailian Guo, Fang Fang, Peifang Xiao, Linbo Cai, Gen Li, Yang Yang, Zimu Zhang, Yanfang Tao, Li Gao, Zhizhuo Du, Ying Yang, Fenli Zhang, Yijun Wu, Zhongling Wei, Yongping Zhang, Yixin Hu, Yizhen Li, Shaoyan Hu, Jian Pan, Jianqin Li, Hailong He, Zhiheng Li

**Affiliations:** 1 Department of Hematology and Oncology, Children’s Hospital of Soochow University, Suzhou, 215000 Jiangsu China; 2 Institute of Pediatric Research, Children’s Hospital of Soochow University, Suzhou, 215000 Jiangsu China; 3 Department of Traditional Chinese Medicine, Children’s Hospital of Soochow University, Suzhou, 215003 Jiangsu China; 4 Department of Pediatrics, Affiliated Hospital of Guizhou Medical University, Guiyang, Guizhou, 550000 China

**Keywords:** AMKL, BRD4, GNE-987, Apoptosis, PIM1

## Abstract

**Background:**

Acute megakaryoblastic leukemia (AMKL), a rare but aggressive subtype of acute myeloid leukemia (AML), currently has limited treatment options. Given the emerging role of super-enhancers (SEs) in governing oncogenic transcriptional programs, we sought to investigate BRD4 - a key reader of SE-associated chromatin - as a potential therapeutic target in AMKL, along with identifying critical SE-regulated genes essential for AMKL survival.

**Methods:**

Genetic inhibition and pharmacological degradation of BRD4 using GNE-987 were performed to assess its impact on AMKL cell proliferation and apoptosis. Through integrative multi-omics analysis, H3K27ac ChIP-seq, RNA-seq, and BRD4 CUT&TAG assays were incorporated to identify SE-associated oncogenes in AMKL. Functional validation of PIM1 genetic ablation in vitro and in vivo were performed to evaluate its role in AMKL cell viability and leukemic burden.

**Results:**

BRD4 expression was significantly elevated in AMKL compared to other AML subtypes. Genetic inhibition or pharmacological degradation of BRD4 by GNE-987 potently suppressed AMKL cell proliferation and induced apoptosis, confirming BRD4 as a critical dependency in AMKL. Through integrative multi-omics analysis incorporating H3K27ac ChIP-seq, RNA-seq and BRD4 CUT&TAG, we identified PIM1 as a critical SE-associated oncogene directly regulated by BRD4 in AMKL. Importantly, PIM1 demonstrated significantly higher essentiality in AMKL compared to other AML subtypes. Functional validation revealed that PIM1 genetic ablation profoundly impaired AMKL cell viability in vitro and reduced leukemic burden in vivo.

**Conclusion:**

Our work elucidates the BRD4-PIM1 axis as a critical driver of AMKL pathogenesis, wherein BRD4 maintains high PIM1 expression through SE-mediated transcriptional regulation. These findings provide a rationale for targeting BRD4 or PIM1 in precision therapy for AMKL.

**Supplementary Information:**

The online version contains supplementary material available at 10.1186/s12935-026-04377-1.

## Key implications

BRD4 inhibition represents a promising therapeutic avenue for AMKL.

PIM1 is a BRD4-regulated super-enhancer-associated oncogene in AMKL.

The BRD4-PIM1 axis offers a molecular framework for understanding AMKL pathogenesis.

**Supplementary Information:**

The online version contains supplementary material available at 10.1186/s12935-026-04377-1.

## Introduction

Acute megakaryoblastic leukemia (AMKL) is an acute myeloid leukemia (AML) subtype characterized by the presence of blasts exhibiting aberrant megakaryocytic differentiation [1, 2]. The occurrence of AMKL is age-biased which accounting for 4–15% of childhood AML cases but only ~ 1% of adult AML cases [1]. AMKL was driven by distinct oncogenic fusions or gene mutations, leading to heterogeneous clinical outcomes [3]. While Down syndrome-associated AMKL (DS-AMKL) has a relatively favorable prognosis, non-DS-AMKL patients face poor survival rates. This heterogenous nature of AMKL posted great challenge to the clinical treatment. Therefore, elucidating the pathogenesis of AMKL and identifying common mechanistic pathways could reveal novel therapeutic targets and improve clinical management.

Studies have revealed that aberrant epigenetic regulation plays a central role in disease pathogenesis. In particular, the abnormal activation of super enhancers (SEs), which are genomic regions densely enriched with histone modifications such as histone H3 lysine 27 acetylation (H3K27ac), play a critical role in maintaining tumor cell identity and oncogenic signaling pathways [4, 5]. Our group and others have mapped the SE landscape in AML [6]. In the context of AMKL, it is reported that ETO2-GLIS2 + AMKL showed distinct enhancer program and a pronounced dependent on SE-KIT activity [7], highlighting a unique super-enhancer vulnerability in this leukemia subtype. BRD4, a key member of the BET protein family and an established reader of H3K27ac, emerges as a pivotal regulator of SE-mediated transcription. By recognizing acetylated histones and recruiting transcriptional machinery, BRD4 amplifies the transcription of SE-related oncogenes. This positions BRD4 as a potential therapeutic target to disrupt SE-driven oncogenic program. BRD4 was identified as therapeutic target of AML by RNAi screen [8], but its functional role and therapeutic potential in the AMKL subtype remain to be fully elucidated.

Small-molecule inhibitors targeting BRD4 (e.g., JQ1 or OTX015) competitively bind to its bromodomain, disrupting BRD4-chromatin interactions and selectively suppressing SE-dependent oncogenic transcription programs [9]. More recently, Proteolysis-targeting chimeras (PROTAC)-based BRD4 degraders (e.g., ARV-825, GNE-987) have emerged as a promising alternative strategy, achieving potent and sustained effects through targeted protein degradation [10]. Preclinical and clinical studies have demonstrated their remarkable efficacy across various malignancies, including hematological malignancies [11] and solid tumors [12–14], exhibiting potent cell cycle arrest and apoptosis induction. Here, we comprehensively investigated the role of BRD4 in AMKL by using the PROTAC inhibitor GNE-987 and identified AMKL-specific super-enhancers (SEs) that are critical for leukemia cell survival.

## Methods

### Cell culture

All human AML cell lines were obtained from Shanghai Zhong Qiao Xin Zhou Biotechnology Co., Ltd. (Shanghai, China) within 5 years. Cells were maintained in RPMI-1640 medium (Gibco, OR, USA) supplemented with 10% fetal bovine serum (VivaCell, China) and 1% penicillin-streptomycin (Beyotime, China) at 37 °C/5% CO₂. 10ng/mL GM-CSF (Novoprotein, China) was supplemented for M07E maintenance. Cell identity was authenticated by short tandem repeat (STR) profiling.Mycoplasma testing was routinely performed to avoid contamination.

### Reagent

GNE-987, ARV-825, JQ1, and TP-3654 were purchased from MedChemExpress company (NJ, USA). Antibodies used in this study were as follows: anti-Caspase-3 (9661 S, CST, MA, USA), anti-PARP (9542 S, CST, MA, USA), anti-GAPDH (AP0063, Bioworld Technology Inc.), anti-BRD2 (5848 S, CST, MA, USA), anti-BRD3 (11859-1-AP, Proteintech, IL, USA), anti-BRD4 (13440 S, CST, MA, USA), anti-C-Myc (9402 S, CST, MA, USA), anti-VHL (68547 S, CST, MA, USA), anti-Pim1(394600, Thermo Fisher, MA, USA) and anti-human Ki67 (GB12114, Servicebio, China).

### Cell proliferation assay

Cell counting kit (CCK)-8 assay was used to determine the cell proliferation ability. To test the effect of drug treatment on AMKL cell survival, 2,000 AMKL cells were plated into 96-well plate and incubated with serial concentration of drug for 72 h, with cells treated with DMSO as control. To determine the impact of gene of interest on AMKL cell proliferation, cells transduced with shRNA or shNC were seeded into the 96-well plate at a density of 2 × 10^3^ cells/well. CCK-8 assay (APExBIO, K1018, TX, USA) was performed after 2, 4, 6, 8 days of culture. The absorbance at 450nM was measured by a spectrometer (Thermo Fisher, MA, USA). Data were presented as the mean ±standard deviation (SD) from three independent experiments. The half-maximal inhibitory (IC50) concertation was obtained using the Graphpad Prism software (San Diego, CA, USA).

### Cell cycle analysis

Cell cycle analysis was performed using the cell cycle detection kit (Beyotime, China). In brief, cells were harvested after treated with drugs for indicated time and fixed with 70% ethanol at 4℃ overnight. On the next day, cells were stained with propidium iodide(PI) and RNase A at 37℃ for 30 min in the dark. Cell cycle distribution were subsequently determined by flow cytometry on the DeFLEX flow cytometer (Beckman Coulter, FL, USA) and analyzed by FlowJO software (OR, USA).

### Cell apoptosis

Cell apoptosis was accessed by flow cytometry using the FITC-annexin V/PI staining kit (BD Biosciences, CA, USA). Briefly, cells were collected and washed by 1 x binding buffer. Subsequently, cells were stained with FITC-Annexin V antibody and PI solution for 30 min in the dark at room temperature. After washed with binding buffer, cells were resuspended in fresh buffer and analyzed immediately by flow cytometry. Data analysis was performed using FlowJo software (OR, USA).

### Lentivirus-mediated gene overexpression and silence

Lentiviral particles were produced by transfecting 293FT cells with a lentiviral transfer plasmid (containing the gene of interest for overexpression or shRNA for gene silencing) along with packaging plasmids, which were psPAX2 and pMD2.G (Addgene MA, USA), using a polyethylenimine (PEI, Sigma-Aldrich, MO, USA) transfection reagent. After 48–72 h post transfection, the lenti-viral supernatant was collected, filtered through a 0.45 μm PVDF filter. For transduction, AMKL cells are incubated with the viral supernatant in the presence of polybrene (1 µg/mL, Sigma-Aldrich, MO, USA) to enhance transfection efficiency, followed by selection with antibiotics (1ug/mL puromycin, Beyotime, China) after 48–72 h post transfection. The overexpression or silence of gene was confirmed by real-time PCR and Western blot. The sequence of shRNA used in this study were listed in Supplementary Table e1.

### CRISPR/dCas9-mediated enhancer inactivation

M07E cells stably expressing the catalytically inactive dCas9 protein were established by lentiviral transduction, followed by selection with blasticidin (10 µg/mL) for 7 days. Subsequently, lentiviral particles harboring sgRNAs targeting enhancer regions E1 or E2, or a empty vector control, were transduced into dCas9-expressing M07E cells. Cells were then selected with puromycin (1 µg/mL) for 3 days to enrich for successfully transduced populations. Prepared lentivirus were obtained from GENECHEM (Shanghai, China). The sequences of sgRNAs targeting E1 and E2 are provided in Supplementary Table 1.

### AMKL mice model development

CMK cells expressing luciferase were used to establish the in vivo AMKL model. Cells transfected with shControl or shBRD4/PIM1 (1x 10^6^/mice) were in intravenously transplanted into 6–8-week NSG mice (Shanghai Model Organisms Center, Inc, Shanghai, China) Bioluminescence imaging was performed weekly to evaluate the disease burden using the imaging system (Berthold, NightOWL II LB 983). Mice body weight and activity were monitored through the whole experiment. Upon reaching humane endpoint criteria, mice were euthanized via CO₂ asphyxiation followed by cervical dislocation. Tissues including bone marrow, spleen and liver were collected for either flow cytometry analysis or histopathological examination to evaluate the leukemia infiltration.

### RNAseq analysis

RNA-Seq analysis was performed by Novogene Bioinformatics Technology Co., Ltd. (Beijing, China). Differentially expressed genes (DEGs) were identified using DESeq2 analysis (|log2FoldChange| > 1.0 and adjusted-*p* < 0.05). Enrichment analysis was conducted using GSEA software, which was jointly developed by UC San Diego and the Broad Institute, to obtain a deeper understanding of the biological processes linked to the differentially expressed genes (DEGs).

### ChIP sequencing and super-enhancer prediction

AMKL cells (50 million) were collected and fixed with 1% paraformaldehyde to cross-link target proteins to DNA. The cross-linking was quenched with 0.125 M glycine, followed by cell lysis using lysis buffer to release chromatin. The chromatin was then fragmented into 200–800 bp pieces via sonication. The samples were divided into input and antibody-captured (H3K27ac) groups, with the H3K27ac group incubated with H3K27ac antibody for antigen-antibody conjugation. The next day, Dynabeads Protein G beads were added to the samples for immunoprecipitation. After washing, the cross-links were reversed by overnight incubation with 5 M NaCl at 65 °C in a metal bath. Finally, the released DNA fragments were purified for subsequent high-throughput sequencing analysis. In the present study, raw data of ChIP-Seq H3K27ac datasets were aligned to UCSC hg38 (the reference genome) with Bowtie2 (v 2.4.1) [15]. Peaks were called with MACS2 (v2.0.10). The bigwig files for these datasets were subsequently visualized using Integrative Genomics Viewer (IGV) [16]. Then, we identified super enhancers (SEs) by the ROSE (Rank Order of Super Enhancers) method with the parameters -s 12,500 -t 2000 (-s, stitching distance; -t, TSS exclusion zone size) [17].

### Cleavage under targets and tagmentation (CUT & TAG)

CUT & TAG were performed using the Hyperactive Universal CUT&Tag Assay Kit for Illumina Pro kit (Vazyme, China). In brief, 5x10^5^AMKL cells were permeabilized using digitonin-containing wash buffer (containing 20 mM HEPES pH 7.5, 150 mM NaCl, 0.5 mM spermidine, 0.05% digitonin and protease inhibitors) to allow antibody access while maintaining nuclear integrity. After blocking with 2% BSA in Dig-wash buffer, cells are incubated with BRD4 antibody overnight at 4 °C. The second day, cells are washed and incubated with secondary antibody for 1 h at room temperature. The Protein A/G-Tn5 transposome complex, pre-loaded with end adapters, is then added and allowed to bind for 1 h at 4 °C. After extensive washing to remove unbound Tn5, tagmentation is initiated by adding 10 mM MgCl₂ and incubating at 37 °C for 1 h. The reaction is terminated with 10 mM EDTA, 0.1% SDS, and 3 µg Proteinase K, followed by incubation at 55 °C for 1 h to reverse crosslinks. DNA is purified using DNA Extract beads and amplified by PCR for 14 cycles. Library quality is assessed by Bioanalyzer before sequencing on Illumina platforms. The raw data from the CUT&Tag experiments were aligned to the UCSC hg38 reference genome using Bowtie2 (v 2.4.1). Peaks representing regions of interest were identified using MACS2 (v2.0.10).

### Public dataset usage

BRD4 and PIM1 expression across AML subtypes was analyzed using the TCGA-LAML dataset based on the UALCAN database (https://ualcan.path.uab.edu/index.html). Differential expression between AML samples (TCGA-LAML) and normal whole blood controls (GTEx) was assessed via the Gepia2 database (http://gepia2.cancer-pku.cn/). BRD4 dependency in M07E and CMK cells was evaluated using the DepMap portal (https://depmap.org/portal/). Gene expression correlation was determined within the TCGA-LAML cohort.

### Statistical analysis

All experiments were independently repeated at least three times. Statistical analyses were conducted using GraphPad Prism 8.3.0 (GraphPad Software, Inc., CA, USA). For comparisons between two groups, a two-tailed paired Student’s t-test was applied, while one-way ANOVA was used for multiple groups. Results are presented as means ± standard deviation (SD), with statistical significance set at *p < 0.05, **p < 0.01, ***p < 0.001, and ****p < 0.0001.

## Results

### BRD4 is a potential target for AMKL

**Fig. 1:**
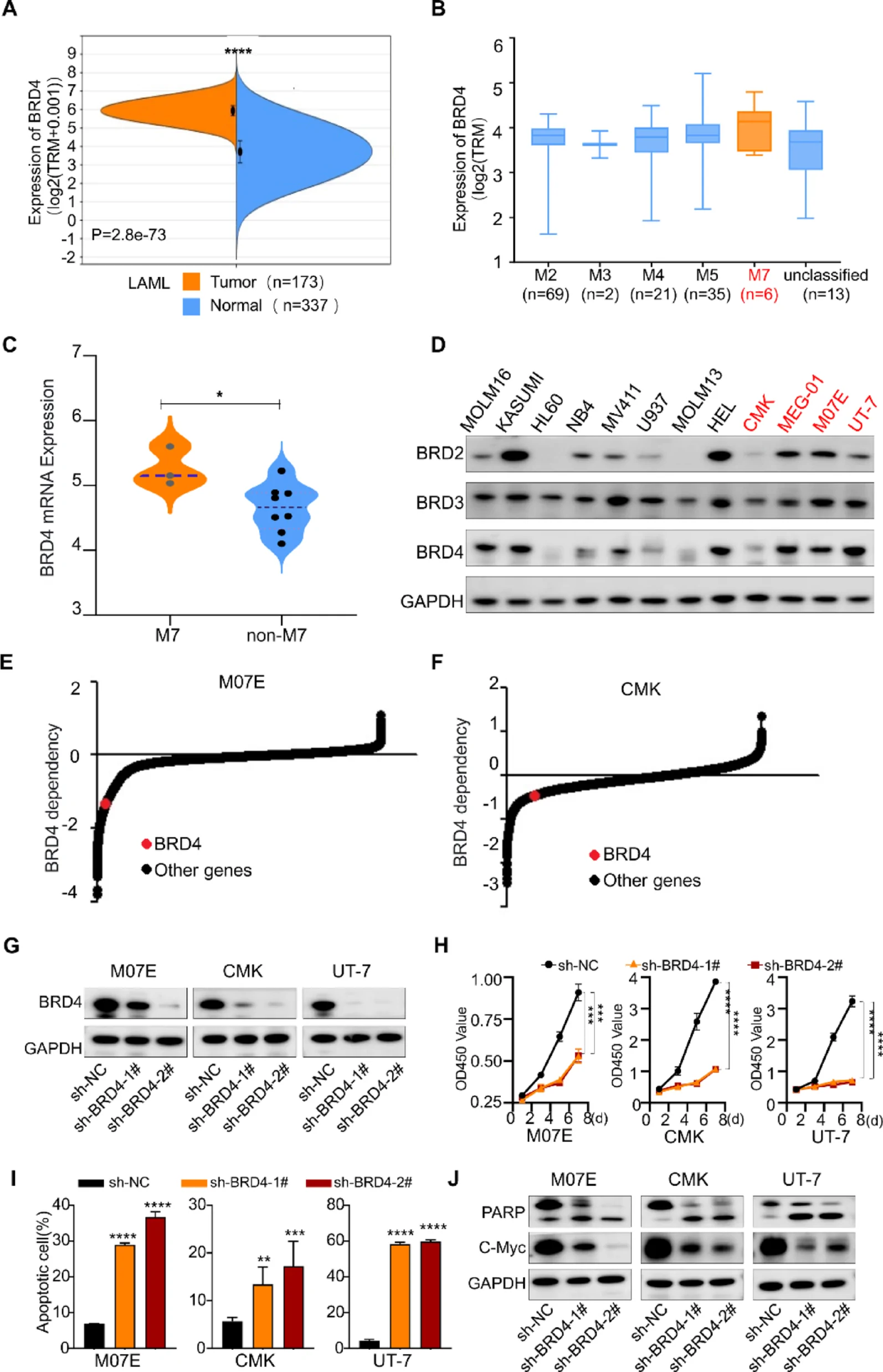
BRD4 is a potential target for AMKL. (**A**) BRD4 mRNA expression in AML patients (TCGA-LAML dataset) versus normal whole blood from healthy donors (GTEx data) generated via GEPIA2.database (http://gepia2.cancer-pku.cn/assets/html/profile.html). (**B**) BRD4 expression across AML subtypes, based on RNAseq of diagnostic AML blasts from our institutional cohort. (**C**) BRD4 expression in AML-M7 and AML-non-M7 patients. Data was generated from the UALCAN website using the TCGA-AML dataset (https://ualcan.path.uab.edu/). (**D**) Protein level of BRD2, BRD3 and BRD4 across AML subtypes. Four M7 cell lines are indicated in red. (**E**-**F**) Analysis of BRD4 dependency in M07E and CMK cell lines, based on data from the DepMap database. (**G**) Western blot confirmation of BRD4 knockdown efficiency in three AMKL cell lines. (**H**) BRD4 knockdown decreased proliferation rates in three AMKL cell lines. (**I**) BRD4 knockdown induced cellular apoptosis in three AMKL cell lines. (**J**) BRD4 knockdown triggered PARP cleavage and reduced c-MYC expression in three AMKL cell lines. Data are representative of ≥ 3 independent experiments (mean ± SD). Statistical significance: *p < 0.05, **p < 0.01, ***p < 0.001

We first investigated the expression level of BRD4 in AML patients. By using the TCGA-LAML cohort from the GEPIA public database, we found BRD4 was overexpressed in AML patients compared with normal samples (*P* = 2.8e-73, Fig. 1A). The BRD4 expression level in AML subgroups based on FAB classification was further analyzed using the pediatric AML cohort from our hospital. Our cohort was composed of 146 pediatric AML patients in which included 6 AMKL patients (FAB-M7). Among different FAB subtypes, AMKL patients showed the highest mRNA expression level of BRD4 (Fig. 1B). This finding was confirmed in the TCGA-LAML cohort, in which FAB-M7 patients exhibited significantly higher BRD4 expression than non-M7 AML patients (Fig. 1C & Supplementary Fig. 1A). High BRD4 expression correlated with inferior prognosis of AML (Supplementary Fig. 1B). Western blot analysis showed that the protein of BET family, which were BRD2, BRD3 and BRD4, were broadly expressed among 12 AML cell lines, including 4 AMKL cells (Fig. 1D). These results indicates that BRD4 is highly expressed in AMKL.

To explore the role of BRD4 in AMKL cells, we used the DepMap database and found that the cell survival of CMK and M07E cells were highly dependent on BRD4 (Fig. 1E-F). Consistent with this dependency, shRNA-mediated knockdown of BRD4 (Fig. 1G) significantly inhibited proliferation in three AMKL cell lines (Fig. 1H). Flow cytometry analysis revealed that BRD4 depletion induced cell apoptosis (Fig. 1I and Supplementary Fig. 2). Furthermore, western blot analysis confirmed the impact of BRD4 on cell growth and apoptosis, demonstrating increased PARP cleavage and reduced c-Myc protein levels (Fig. 1J). These findings suggested BRD4 is essential in AMKL cell survival and could be a promising target of AMKL.

### Depletion of BRD4 delays leukemia development in AMKL mice

**Fig. 2:**
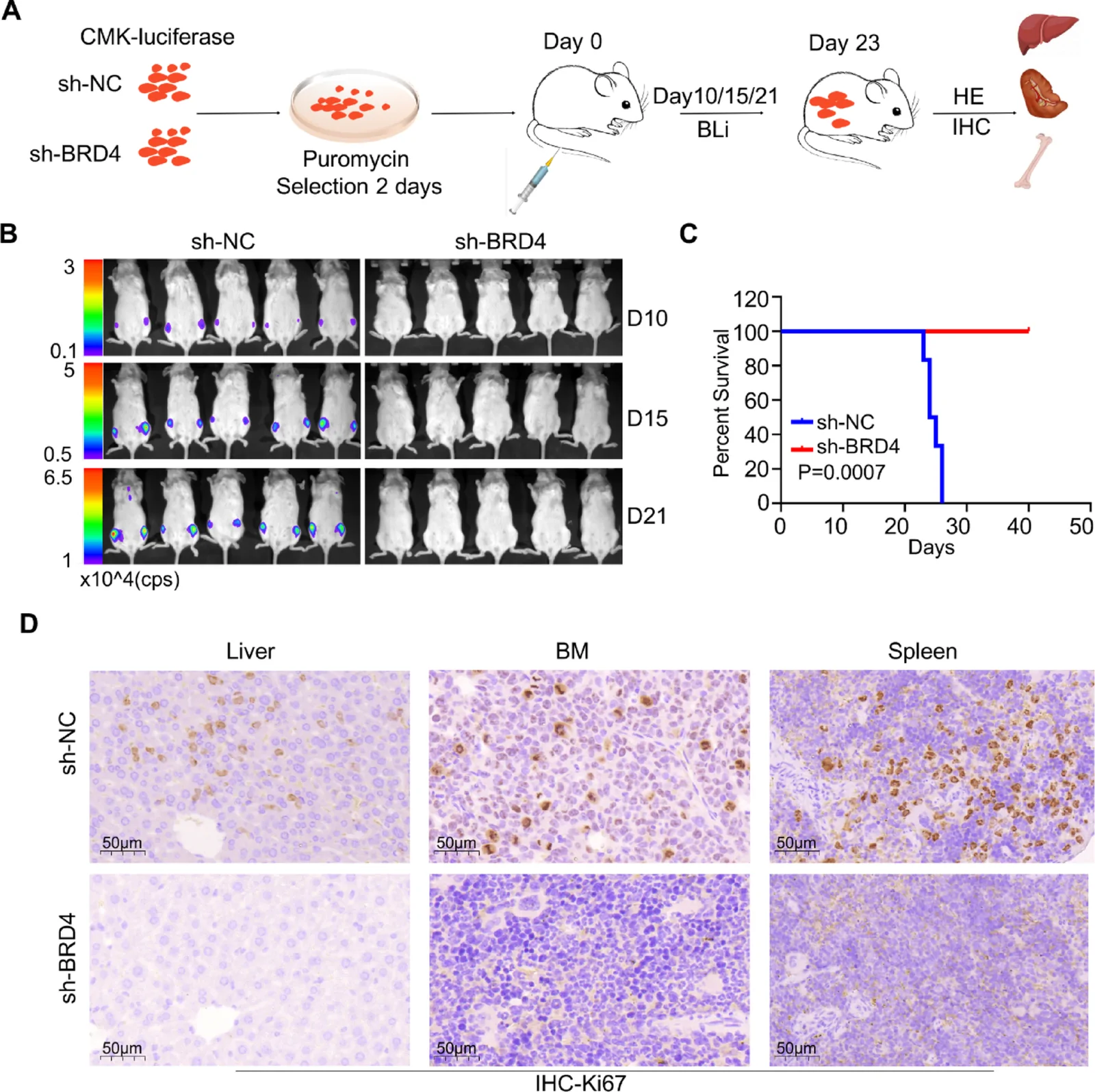
Depletion of BRD4 delays leukemia development in AMKL mice. (**A**)The scheme of assessing the impact of BRD4 knockdown in the CMK AMKL mouse model. BLi: bioluminescence imaging. (**B**) Representative images showing bioluminescence in AMKL mice at indicated days post-transplantation. (**C**) BRD4 knockdown prolonged survival in CMK AMKL mice. (**D**) Reduced leukemic infiltration in bone marrow, liver, and spleen confirmed by Ki67 IHC staining in CMK-shBRD4 versus control mice. Scale bar represents 50 μm

To investigate the in *vivo* role of BRD4 in AMKL development, Luciferase-expressing CMK cells transduced with BRD4 shRNAs or control shRNAs were intravenously injected into NSG mice. Bioluminescent imaging was routinely performed to monitor the disease progression (Fig. 2A). Depletion of BRD4 by shRNA2 resulted in delayed AMKL progression and prolonged survival time of AMKL mice (Fig. 2B-C, Supplementary Fig. 3A). At the day of sacrifice, leukemic burden in bone marrow, liver and spleen were assessed by bioluminescent imaging and flow cytometry analysis of human CD45 + cells. The results revealed reduced leukemia burden in shBRD4 group compared with the control shRNA group (Supplementary Fig. 3B-E). This reduction in leukemic infiltration was further confirmed by H&E staining and IHC for human Ki67 on tissue sections from mice transplanted with shBRD4 CMK cells (Fig. 2D, Supplementary Fig. 4). Together, these results demonstrate that BRD4 inhibition suppresses AMKL progression in vivo.

### BRD4 PROTAC inhibitor GNE-987 induces cell death in AMKL cells

**Fig. 3:**
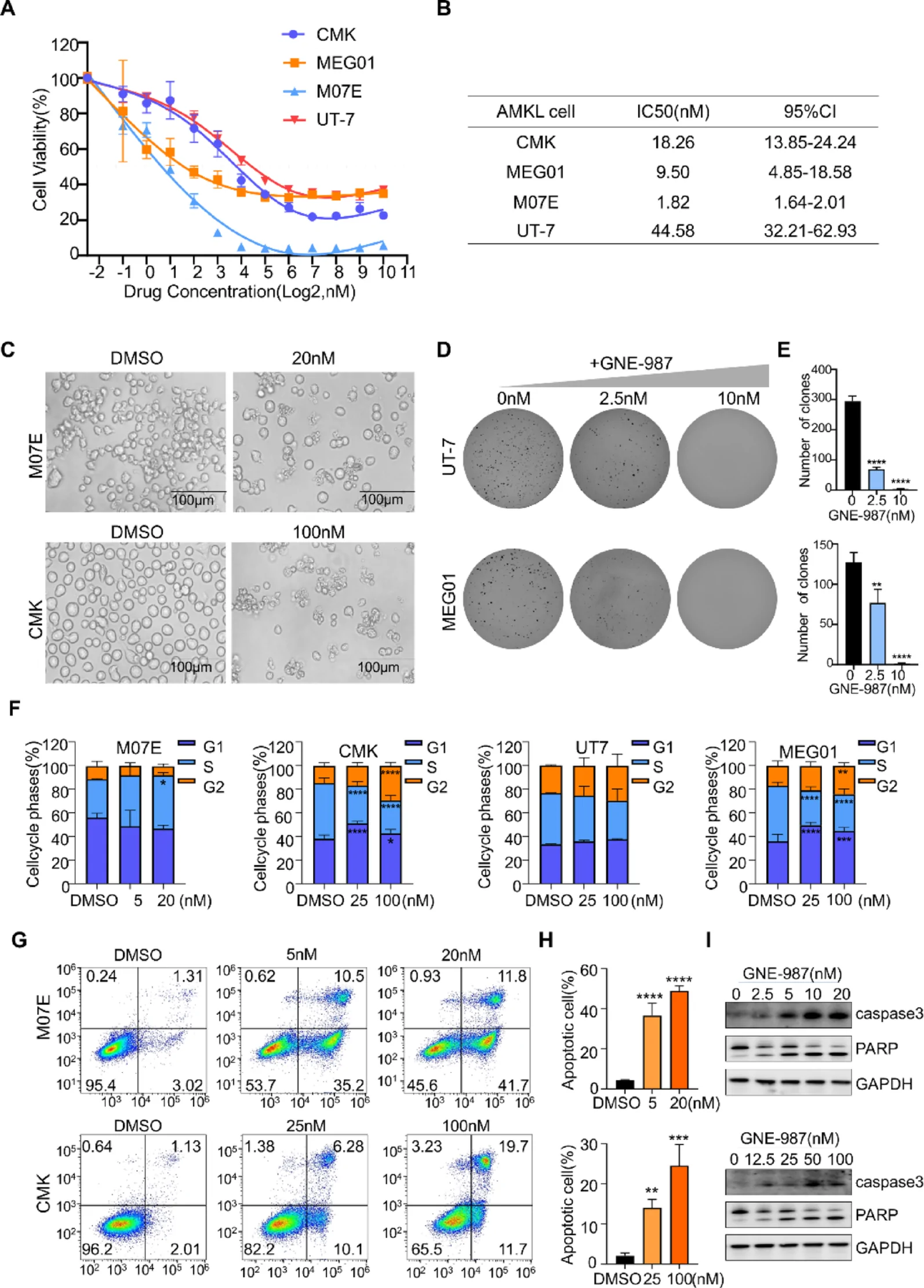
BRD4 PROTAC inhibitor GNE-987 induces cell death in AMKL cells. (**A**)The BRD4-targeting PROTAC GNE987 inhibits growth in AMKL cell lines. (**B**) IC50 of GNE987 in four AMKL cell lines. (**C**) Morphological changes in M07E and CMK cells treated with GNE987 versus vehicle (DMSO). (**D**) GNE987 treatment reduced colony formation capacity in UT7 and MEG01 cells. (**E**) Quantification of colony numbers in UT7 and MEG01 cells treated with GNE987 versus vehicle (DMSO). (**F**) GNE987 treatment altered cell cycle distribution in four AMKL cell lines. (**G**) Flow cytometry analysis of apoptosis in GNE987-treated M07E (upper) and CMK (lower) cells. (**H**) Quantification of apoptotic cells of GNE987-treated M07E (upper) and CMK (lower) cells. (**I**) GNE987 treatment induced cleavage of capase3 and PARP in M07E (upper) and CMK (lower) cells

We next evaluated the efficacy of GNE-987—a BRD4-targeting PROTAC degrader—in AMKL cell lines. All four AMKL cell lines exhibited high sensitivity to GNE-987, with IC₅₀ values below 100 nM (Fig. 3A-B). Treatment with GNE987 for 48 h inhibited cell growth in a dose-dependent manner and induced significant cell death (Fig. 3C). Consistent with this, colony formation assays revealed a substantial reduction in the colony-forming ability of AMKL cells upon GNE-987 treatment (Fig. 3D-E). Cell cycle analysis demonstrated that GNE-987 disrupted cell cycle progression in all four AMKL cells (Fig. 3F). Furthermore, GNE-987 treatment increased the proportion of early- and late-apoptotic cells (Fig. 3G-H). The pro-apoptotic effect was confirmed by the induction of caspase-3 and PARP cleavage (Fig. 3I). Therefore, these results indicate that targeting BRD4 with GNE-987 effectively suppresses AMKL cell survival by inducing apoptosis.

**Fig. 4:**
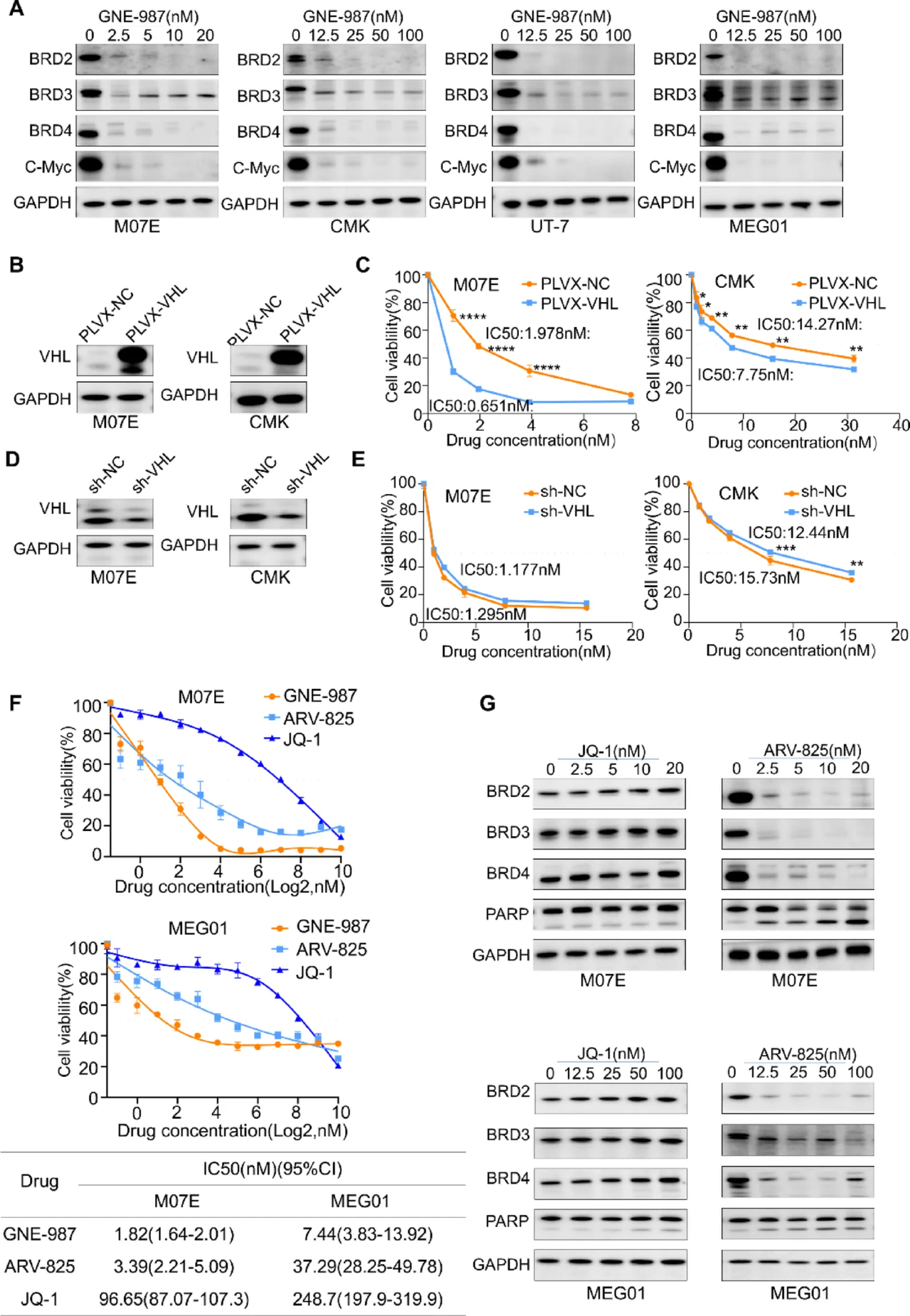
GNE987 induces BET protein degradation through VHL-mediated mechanisms. (**A**) GNE987 treatment induced degradation of BRD2, BRD3, and BRD4, and reduced c-Myc expression in four AMKL cell lines. (**B**) VHL overexpression in M07E (left) and CMK (right) cells. (**C**) VHL overexpression sensitized M07E (left) and CMK (right) cells to GNE987. (**D**) Western blot confirmation of VHL knockdown efficiency. (**E**) Knockdown of VHL increased the IC50 of GNE987 in M07E (left) and CMK (right) cells. (**F**) Comparative efficacy of three BRD4 inhibitors (GNE987, ARV-825, and JQ1) in M07E (upper) and MEG-01 (lower) cells. IC50 values are summarized in the table. (**G**) Differential effects of BET inhibitors (JQ1 and ARV-825) on BET protein levels

GNE987 was designed as a chimera which couples a BRD4-targeting warhead to a VHL (Von Hippel-Lindau) E3 ligase ligand, thereby enabling targeted ubiquitination and proteasomal degradation of BET family [9]. Consistent with its design, GNE-987 induced potent depletion of BET proteins across all four tested AMKL cell lines at nanomolar concentrations (Fig. 4A). Mechanistic studies confirmed the VHL-dependence of this degradation. Overexpression of VHL in M07E and CMK cells significantly increased IC₅₀ values (Fig. 4B-C), while VHL knockdown via shRNA enhanced sensitivity to GNE-987 (Fig. 4D-E). These results demonstrate that efficacy of GNE-987 is directly correlated with cellular VHL expression levels.

We conducted a comparative analysis of BRD4-targeting agents in AMKL cells, evaluating the early-generation inhibitor JQ1 alongside two PROTAC degraders (ARV-825 and GNE-987). GNE-987 demonstrated superior potency, showing the lowest IC50 values in both M07E and MEG-01 cell lines, while JQ1 exhibited the weakest cytotoxic effects (Fig. 4F). As expected, both PROTAC molecules effectively induced BET protein degradation, whereas JQ1 treatment showed no degradation activity (Fig. 4G), which is consistent with its competitive binding mechanism. These results highlight the therapeutic advantage of PROTAC-mediated degradation over conventional BRD4 inhibition in AMKL.

### PIM1 is a target of BRD4 in AMKL cells

**Fig. 5:**
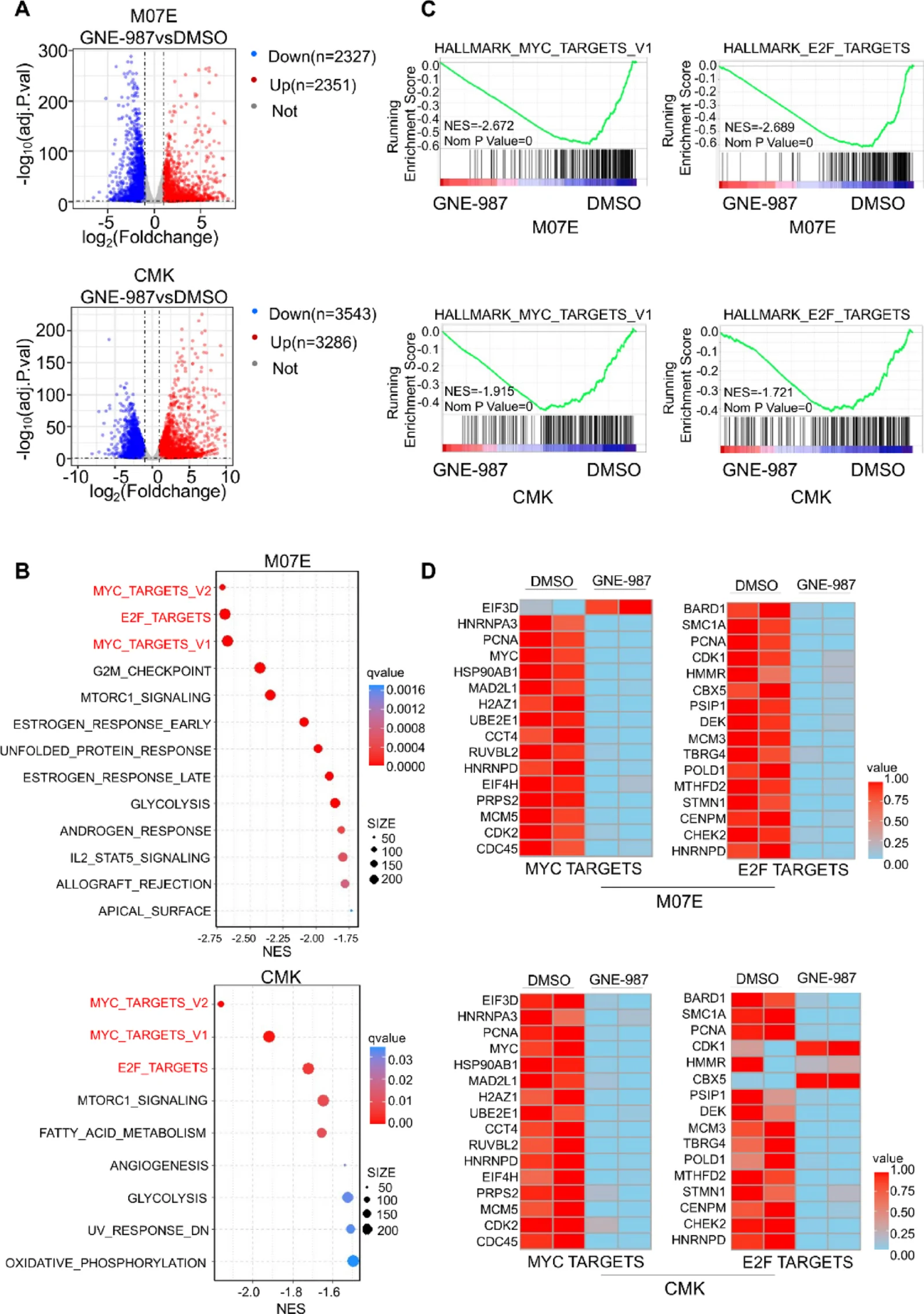
BRD4 inhibition disrupts MYC and E2F transcriptional programs. (**A**) Volcano plots display up- and down-regulated genes in M07E (upper) and CMK (lower) cells after GNE987 treatment (|log2FoldChange| > 1.0 and adjusted-*p* < 0.05). (**B**) Gene Set Enrichment Analysis (GSEA) identifies biologic processes (*p* < 0.05) enriched in GNE987-mediated transcriptional changes. (**C**) GSEA plots demonstrate significant downregulation of MYC and E2F target pathways in GNE987-treated M07E (upper) and CMK (lower) cells. (**D**) Heatmaps depicting representative differentially expressed genes from MYC and E2F target pathways in GNE987-treated M07E (upper) and CMK (lower) cells

To further elucidate the molecular mechanism of GNE-987-induced cell apoptosis in AMKL cells, we performed transcriptome analysis by RNA sequencing in M07E and CMK cells treated with GNE-987. Compared with the parental cells, GNE-987 drastically altered the transcriptome of both cell lines, with 2,351 genes significantly upregulated and 2,327 genes down-regulated in M07E cells, and 3,286 up-regulated genes and 3,543 down-regulated in CMK cells upon GNE-987 treatment (Fig. 5A, supplementary Tables 2–3). GSEA analysis revealed that GNE-987-mediated deregulated genes were consistently enriched in the “MYC-targets” and “E2F-targets” genesets in both M07E and CMK cells (Fig. 5B-C). Representative genes of these genesets were listed in the Fig. 5D. The regulation on MYC was confirmed by western blot showing that c-MYC protein was significantly down-regulated after treated with GNE-987 in AMKL cell lines (Fig. 4A).

**Fig. 6:**
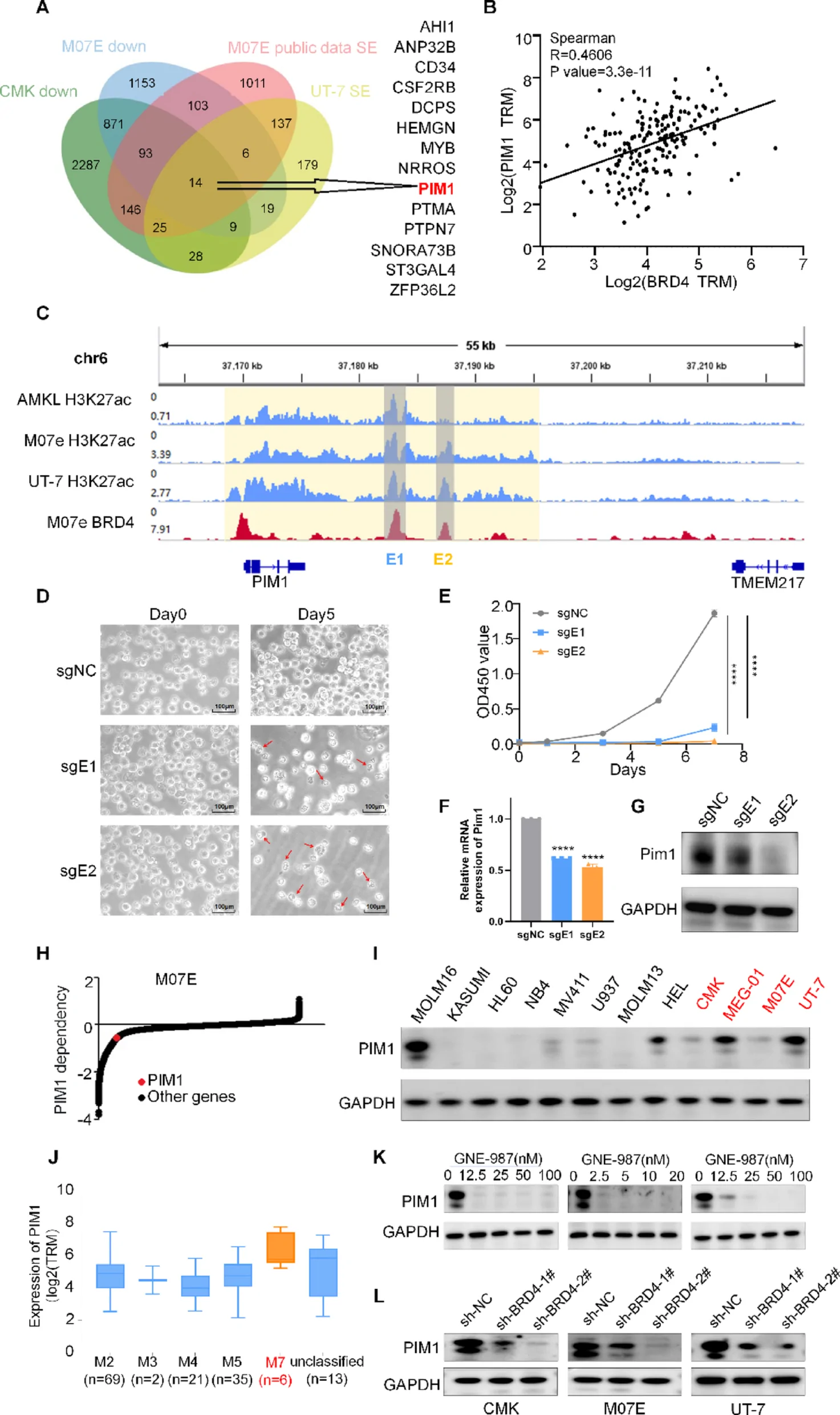
Identification of PIM1 as a BRD4 target in AMKL. (**A**) 14 candidate genes identified by intersection of BRD4-suppressed genes (CMK/M07E) and super-enhancer-marked genes (UT7/M07E H3K27ac ChIP-seq). (**B**) Positive correlation between PIM1 and BRD4 expression in TCGA-LAML cohort (Pearson *r* = 0.4606, *p* = 3.3e-11. (**C**) IGV plot of H3K27ac marks in primary AMKL, M07E, and UT-7 cells and BRD4 peaks at the PIM1 genomic region. The two potential enhancer regions E1 and E2 were marked in grey. (**D**) Morphology of dCas9-expressing M07E cells on days 0 and 5 after transfection with enhancer sgRNAs or a non-targeting control (sgNC). Red arrows indicate dead cells. Scale bar: 100 μm (**E**) Reduction of cell proliferation rate in M07E-dCas9 cells transfected with enhancer sgRNAs or sgNC. The mRNA (**F**) and protein (**G**) level of PIM1 in M07E-dCas9 cells transfected with enhancer sgRNAs or sgNC on day5. (**H**) PIM1 dependency profile in M07E cells. (**I**) PIM1 expression level in AML cell lines. Four M7 cell lines are indicated in red. (**J**) Differential PIM1 expression by AML subtype. (**K**) Downregulation of PIM1 in GNE987-treated AMKL cells. (**L**) PIM1 suppression following BRD4 knockdown in AMKL cells

BRD4 is a well-established reader of H3K27ac and regulates genes marked by this histone modification, particularly those within super-enhancers (SEs). These SEs play essential roles in cell survival and fate determination. To identify BRD4-dependent SEs critical for AMKL, we first mapped AMKL-specific SEs in two AMKL cell lines (M07E and UT-7) using H3K27ac ChIP-seq data (Supplementary Tables 4–5). Intersecting these AMKL-SEs with genes downregulated by the BRD4 inhibitor GNE-987 in both cell lines identified 14 BRD4-regulated SE-associated genes, including AHI1, ANP32B, PIM1 (Fig. 6A). Among these, PIM1 exhibited the strongest positive correlation with BRD4 expression (*R* = 0.4606; Fig. 6B, Supplementary Fig. 5). Integrative Genomics Viewer (IGV) visualization confirmed robust H3K27ac modification near the PIM1 locus in primary AMKL patient sample (GSE131459) and 2 AMKL cell lines (Fig. 6C). Notably, significant BRD4 binding at PIM1’s promoter was observed (Fig. 6C, Supplementary Table 6), implying PIM1 as a direct, SE-regulated target of BRD4 in AMKL cells.

Beyond the canonical promoter-proximal SE, we identified two distinct H3K27ac-enriched regions accompanied by robust BRD4 occupancy in the 3′ downstream region of the PIM1 locus, suggesting the presence of additional functional super-enhancers. To investigate their regulatory role, we employed the CRISPR/dCas9 system to inactivate these two regions named E1 and E2 in M07E cells(Fig. 6C). Cells transduced with sgRNAs targeting either E1 or E2 exhibited marked suppression of proliferation and pronounced cell death (Fig. 6D-E). Importantly, inactivation of these 3′ SEs resulted in significant downregulation of PIM1 expression at both mRNA and protein levels (Fig. 6F-G), confirming that PIM1 transcription is positively regulated by these distal enhancer elements in AMKL cells.

### PIM1 plays an essential role in AMKL survival

**Fig. 7:**
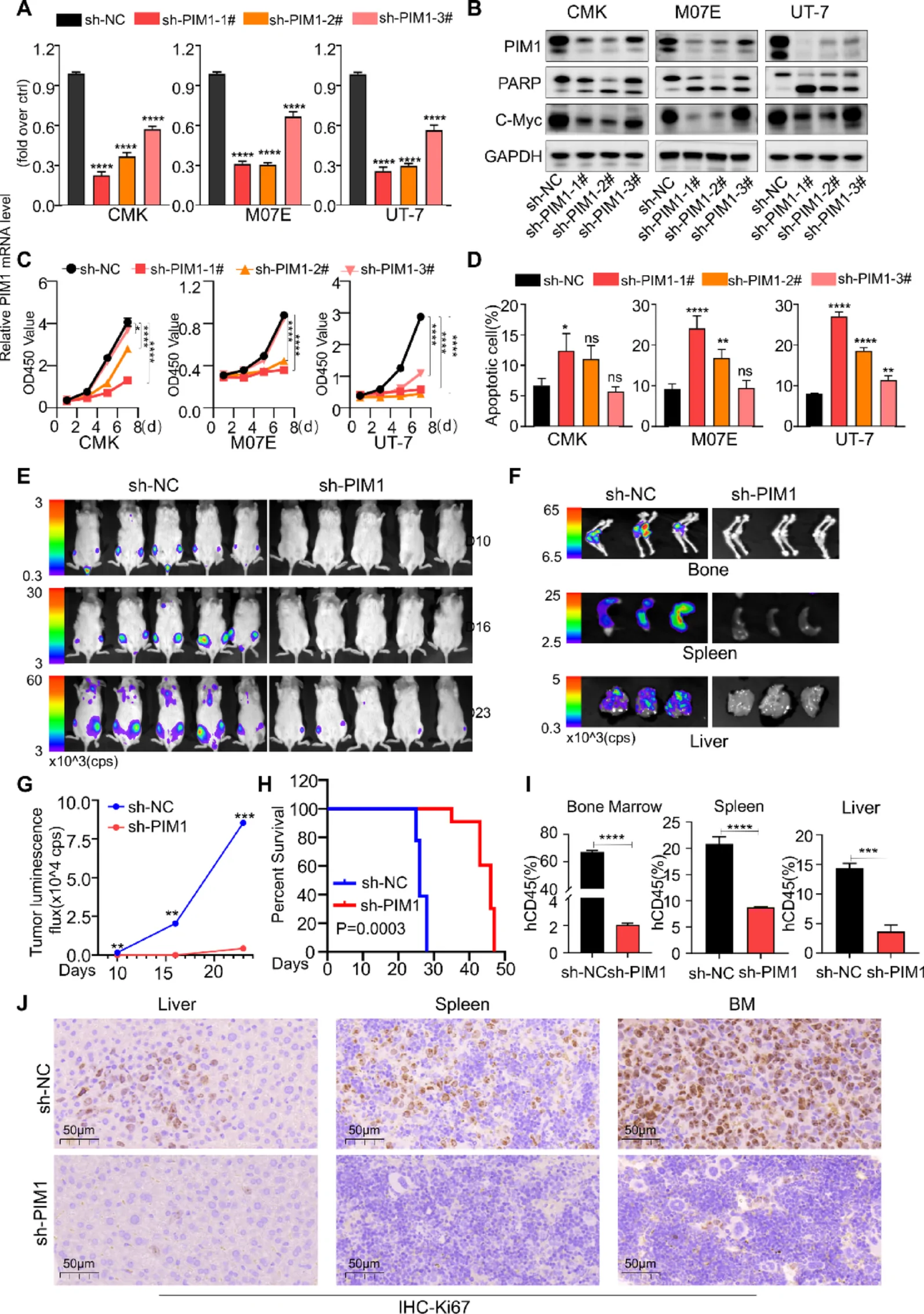
PIM1 plays an essential role in AMKL development. (**A**) PIM1 mRNA knockdown efficiency in 3 AMKL cell lines using shRNA. (**B**) Western blot analysis of PARP cleavage and c-Myc reduction in 3 AMKL cell lines following PIM1 knockdown. (**C**) Reduction of cell proliferation rate in 3 AMKL cell lines following PIM1 knockdown. (**D**) Quantification of apoptotic cells by flow cytometry in 3 AMKL cell lines following PIM1 knockdown. (**E**) In vivo bioluminescence imaging of AMKL mice transplanted with CMK cells stably expressing shNC or shPIM1 (**F**) Bioluminescence intensity in the liver, spleen and femur in AMKL mice at sacrifice. (**G**) Quantification of bioluminescence signals in AMKL mice at indicated days. (**H**) Survival analysis of AMKL mice transplanted with shNC- or shPIM1-transfected CMK cells (**I**) Flow cytometry analysis of hCD45 + leukemia cells in the bone marrow, spleen and liver at sacrifice. (**J**) Immunohistochemical staining of hKi67 in the bone marrow, liver, and spleen of AMKL mice (scale bar: 50 μm)

While PIM1 was identified as a poor prognostic factor in AML [18] and recognized as a pluripotency-associated SE-regulated gene maintaining stem cell pluripotency [19], its role in specific AML subtypes remain poorly characterized. PIM1 was robustly identified as a SE in both AMKL cell lines and primary patient samples (Supplementary Fig. 6A, Supplementary Tables 4, 5, 7), in contrast to its rare SE status in non-M7 AML (Supplementary Fig. 6B, Supplementary Table 8), implicating its critical role in AMKL cell fate determination. Analysis of the DepMap database revealed a high dependency on PIM1 in M07E cells (Fig. 6H). Furthermore, elevated PIM1 expression is specifically in AML FAB-M6 (represented by HEL cells) and FAB-M7 subtypes (Fig. 6I). Consistent with this, within our AML cohort, PIM1 mRNA expression was highest in AMKL compared to other FAB subtypes (Fig. 6J), a finding corroborated by the TCGA-LAML dataset (Supplementary Fig. 7A). PIM1 is highly expressed in AML compared to normal control (*P* = 0.02, Supplementary Fig. 7B). In addition, high PIM1 expression was significantly associated with poor prognosis of AML patients (*P* = 1.8E-5, Supplementary Fig. 7C). These findings position PIM1 as a potential subtype-selective therapeutic target in AMKL. Suppression of BRD4 expression – either pharmacologically with GNE-987 or genetically using BRD4-targeting shRNA – consistently reduced PIM1 protein levels (Fig. 6K-L), validating BRD4’s direct regulation of PIM1. To assess functional role of PIM1, we knocked down its expression in AMKL cell lines using specific shRNAs (Fig. 7A, B). Efficient PIM1 knockdown in sh1 and sh2 groups resulted in significantly reduced cell proliferation and increased apoptosis (Figure C, D, Supplementary Fig. 8). TP-3654 (Nuvisertib) is a selective PIM1 inhibitor approved by FDA for treatment of patients with relapsed or refractory myelofibrosis (MF). Treatment with TP-3654 also induced growth arrest and apoptosis in CMK and MEG01 cells (Supplementary Fig. 9).

The critical role of PIM1 was further demonstrated in vivo. Mice transplanted with CMK cells expressing shPIM1 (sh1) exhibited a significant delay in leukemia progression and prolonged survival (Fig. 7E-H). Reduced leukemic burden and tissue infiltration in these mice were confirmed by flow cytometry and IHC staining (Fig. 7I-J, Supplementary Fig. 10). Together, these data reveal that PIM1, as an SE-regulated gene, played an essential role in AMKL survival.

## Discussion

AMKL, a rare and aggressive subtype of acute myeloid leukemia (AML), carries a significantly poorer prognosis compared to non-M7 AML subtypes [20]. Recent clinical trial data from the Children’s Oncology Group (COG) reveal a 5-year EFS of 37–62% for pediatric AMKL [21], underscoring the critical need for novel therapeutic targets. Emerging evidence highlights frequent dysregulation of epigenetic mechanisms in AMKL [22], positioning epigenetic regulators as promising therapeutic targets. Notably, BRD4, an epigenetic reader that drives oncogenic transcription programs, exhibits significantly higher expression in AMKL (M7) compared to non-M7 AML, suggesting enhanced therapeutic relevance in this high-risk subtype. In this study, we evaluated the role of BRD4 in AMKL using shRNA-mediated knockdown and GNE-987-mediated protein degradation. Our results demonstrate that BRD4 is essential for AMKL cell survival, and its depletion significantly delays disease progression in vivo. These findings not only align with previous reports implicating BRD4 in AML [23] and other malignancies [24, 25], but also further establish BRD4 as a pivotal epigenetic regulator that sustains AMKL oncogenesis, shedding new light on the subtype-specific epigenetic regulatory networks driving this aggressive leukemia.

Genome-wide profiling of SEs has emerged as a powerful tool for dissecting molecular heterogeneity, both across distinct cancer types [26] and within the same disease [5], enabling SE-based molecular subtyping. Using H3K27ac ChIP-seq profiling in AMKL cells, we systematically identified key super-enhancers (SEs) that maintain AMKL cell identity and drive malignant phenotypes. Also, these SE-associated genes provide preferential transcriptional vulnerability to BRD4 inhibition. Our study identified PIM1 as a critical SE-associated gene regulated by BRD4 uniquely in AMKL, distinguishing it from other non-M7 AML subtypes [6]. PIM1 is transcriptionally regulated by JAK/STAT3 pathway [27, 28], which is constitutively activated in AMKL [29]. Notably, pharmacological inhibition of PIM1 significantly attenuated bone marrow fibrosis in Jak2V617F and MPLW515L murine myelofibrosis models [30]-a therapeutically relevant finding given that fibrosis represents a hallmark pathological feature of AMKL. These findings collectively establish PIM1 as a key mediator of AMKL pathogenesis and a promising therapeutic target for this aggressive leukemia subtype.

We further elucidated the mechanism by which BRD4 regulates PIM1 expression via SEs in AMKL cells and identified two functional SE regions in the PIM1 3′ downstream locus, in addition to the classic promoter-proximal SE. dCAS9-mediated inactivation of these 3′ SEs markedly downregulated PIM1 expression and suppressed AMKL cell proliferation, thereby refining the mechanistic details of BRD4 orchestrating PIM1 transcription through multiple cooperative SE elements in AMKL cells. Notably, this BRD4-PIM1 regulatory axis extends beyond cancer. A cross-species analysis has revealed PIM1 as an evolutionarily conserved SE among placental mammals and is occupied by BRD4 to maintain stem cell pluripotency [19]. These findings confirm that the BRD4-PIM1 axis is involved in both physiological epigenetic regulation and AMKL pathogenesis, and further highlighting the unique molecular characteristics of AMKL among AML subtypes.

While several BRD4 inhibitors have entered clinical trials for both solid tumors and hematopoietic malignancies, first-generation compounds (e.g., JQ1, OTX015) demonstrated limited clinical efficacy, largely due to dose-limiting toxicities [31], spurring the development of next-generation strategies such as PROTACs with the potential of enhanced selectivity and potency [32]. In this study, we employed the PROTAC-based BRD4 inhibitor GNE-987, which selectively degrades BET family proteins via recruitment of the VHL E3 ubiquitin ligase. Treatment with GNE-987 induced rapid and potent degradation of BET proteins, leading to significant AMKL cell death. However, our preliminary in vivo studies revealed significant systemic toxicity with GNE-987 (data not shown), which may limit its clinical application—an issue likely reflecting the essential physiological functions of BRD4. Indeed, homozygous BRD4 knockout causes early embryonic lethality, while heterozygotes exhibit severe multi-organ defects [33], highlighting the inherent challenges of direct BRD4 targeting. This limitation, however, underscores the translational potential of our key finding: the AMKL-specific BRD4-PIM1 regulatory axis, with PIM1 serving as a critical downstream effector of BRD4 in AMKL. Notably, the FDA-approved PIM1 inhibitor Nuvisertib (TP-3654), currently in clinical development for myelofibrosis, may offer a promising alternative to direct BRD4 targeting for AMKL treatment.

This study has several limitations: (1) The rarity of AMKL clinical samples constrained our investigation to cell line and animal models, which may not fully recapitulate human disease heterogeneity; (2) While GNE-987 shows potent preclinical efficacy across malignancies, its unproven clinical performance currently may restrict therapeutic application; and (3) Although we validated PIM1’s critical role in AMKL cell survival in vitro, its direct mechanistic contribution to disease-associated fibrosis requires further in vivo validation using patient-derived models and stromal interaction studies.

## Conclusion

This study identifies BRD4 as a promising therapeutic target in AMKL, characterized by its subtype-specific overexpression. Both genetic suppression and PROTAC-mediated degradation (GNE-987) of BRD4 potently inhibit AMKL cell survival and induce apoptosis. Importantly, we identify PIM1 as an AMKL-selective, BRD4-regulated super-enhancer-associated gene essential for AMKL cell survival. These findings establish the BRD4-PIM1 axis as a critical driver of AMKL pathogenesis and provide a rationale for targeted therapies against this pathway.

## Supplementary Information

Supplementary Material 1.

Supplementary Material 2.

Supplementary Material 3.

Supplementary Material 4.

Supplementary Material 5.

Supplementary Material 6.

Supplementary Material 7.

Supplementary Material 8.

Supplementary Material 9.

Supplementary Material 10.

Supplementary Material 11.

Supplementary Material 12.

Supplementary Material 13.

Supplementary Material 14.

Supplementary Material 15.

Supplementary Material 16.

Supplementary Material 17.

Supplementary Material 18.

Supplementary Material 19.

## Data Availability

The sequencing data generated in this study have been deposited in the Gene Expression Omnibus (GEO) database under the following accession numbers: RNA-seq (GSE274441), ChIP-seq (GSE274632), and BRD4 CUT&Tag in M07E cells (GSE274631). Publicly available H3K27ac ChIP-seq data for M07E cells and primary AMKL patient can be found under accession GSE148446 and GSE131459, respectively.
