# Supplementary material for: BRD4 regulation of PIM1 identifies a novel therapeutic vulnerability in acute megakaryoblastic leukemia
Source: Cancer Cell Int. 2026 Jun 17;26:296. doi: 10.1186/s12935-026-04377-1 (PMC13520172; doi:10.1186/s12935-026-04377-1)
Supplement: Supplementary file 1 [file 12935_2026_4377_MOESM11_ESM.docx]

**Supplementary Figure 1**

(A) Comparison of BRD4 expression across AML subgroups. Data was generated from the UALCAN database using the TCGA-LAML dataset. (B) The correlation of BRD4 expression with prognosis of patients with AML.

**Supplementary Figure 2**

(A) Flow cytometry analysis of cell apoptosis following shRNA-mediated BRD4 knockdown in AMKL cell lines.

**Supplementary Figure 3**

(A) Quantification of bioluminescence intensity in CMK-shBRD4 versus control mice at indicated timepoints. (B) Quantification of bioluminescence intensity in liver (left), spleen (middle), and femur (right) in CMK-shBRD4 versus control mice at endpoint. (C) Representative bioluminescence images of liver (upper), spleen (middle), and femur (lower) in CMK-shBRD4 versus control mice at endpoint. (D) Flow cytometry analysis of human CD45+ leukemic cells in liver (upper), spleen (middle), and femur (lower) in CMK-shBRD4 versus control mice at endpoint. (E) Quantification of the proportion of human CD45+ leukemic cell infiltration in liver (upper), spleen (middle), and femur (lower) in CMK-shBRD4 versus control mice at endpoint.

**Supplementary Figure 4**

(A) HE staining of the liver, bone marrow and spleen from AMKL mice transplanted with shNC- or shBRD4-transfected CMK cells. Scale bar = 200 µm (B) Immunohistochemical staining of hKi67 in the bone marrow, liver, and spleen of AMKL mice. Scale bar = 200 µm.

**Supplementary Figure 5**

(A) Correlation analysis of BRD4 expression with candidate genes (from Figure 6A) in the TCGA-LAML cohort.

**Supplementary Figure 6**

(A) Superenhancer profiling of AMKL cells and primary AMKL patient sample. (B) Superenhancer profiling of 11 AML patient samples.

**Supplementary Figure 7**

(A) Comparison of PIM1 expression across AML subgroups. Data was generated from the UALCAN database using the TCGA-LAML dataset. (B) Comparison of PIM1 expression between AML samples (TCGA-LAML dataset) and normal whole blood from healthy donors (GTEx data). Data were generated in Gepia2 database (http://gepia2.cancer-pku.cn/assets/html/profile.html). (C) The correlation of PIM1 expression with prognosis of patients with AML.

**Supplementary Figure 8**

(A) Flow cytometry analysis of cell apoptosis following shRNA-mediated PIM1 knockdown in AMKL cell lines.

**Supplementary Figure 9**

(A) TP-3654 treatment inhibits proliferation in CMK and MEG-01 cell lines. (B) IC50 values of TP-3654 in CMK and MEG01 cell lines. (C) Apoptosis analysis by flow cytometry in TP-3654-treated AMKL cells. (D) Quantification of apoptotic cells in TP-3654-treated AMKL cells. (E) TP-3654 induces PARP cleavage in CMK and MEG-01 cells.

**Supplementary Figure 10**

(A) Flow cytometry analysis of hCD45+ leukemia cells in the bone marrow, spleen and liver in AMKL mice transplanted CMK cells expression shNC or shPIM1. (B) Quantification of bioluminescence intensity in liver, spleen and femur from CMK-shBRD4 versus control mice at sacrifice. (C) HE staining of liver, spleen and femur from CMK-shBRD4 versus control mice.
